# The diagnostic value of computed tomography in the management of patients with acute flank pain: An observational cohort study

**DOI:** 10.1177/03915603261445189

**Published:** 2026-04-30

**Authors:** Samuel Johan Sårheim, Patrick Juliebø-Jones, Øyvind Ulvik

**Affiliations:** 1 Department of Clinical Medicine (K1), University of Bergen, Norway; 2 Helse Bergen HF, Department of Urology, Haukeland University Hospital, Norway

**Keywords:** acute flank pain, urolithiasis, CT, intervention, ureteroscopy

## Abstract

**Introduction:**

The need for immediate CT in patients with acute flank pain is debated. We aimed to study the diagnostic value and therapeutic impact of CT in these patients. Furthermore, to identify factors associated with intervention and determine who would benefit from acute imaging.

**Material and methods:**

A retrospective review was performed of patients with acute flank pain admitted to the emergency department at Haukeland University Hospital between June 2023-June 2024. In total, 289 patients with 319 referrals were included in the study.

**Results:**

A total of 142 women and 147 men, with a median age of 48 years (IQR 35–60) were registered. Acute CT was performed in 197 cases (62%), and a cause for flank pain was determined in 159 (81%). A ureteral stone was detected in 122 (62%). Acute ureteroscopy was performed in 48 (39%) of these, while 27 (22%) were drained with a JJ-stent or nephrostomy tube only. Irrespective of the cause for acute flank pain, 234 patients (73%) received conservative treatment only. Patients needing intervention had higher levels of CRP (52 vs 15 mg/L, *p* < 0.001) and creatinine (108 vs 87 μmol/L, *p* < 0.001) compared to those treated conservatively. 219 patients (69%) had CT at follow-up after 3–4 weeks. A persistent ureteral stone needing ureteroscopy was registered in 40 (19%).

**Conclusion:**

CT in patients with acute flank pain may be reserved for complicated cases with signs of infection, elevated CRP and creatinine levels. In uncomplicated cases, CT can safely be deferred until follow-up after 3–4 weeks.

## Introduction

Acute abdomen, defined as rapid onset abdominal pain with a duration shorter than 7 days, is a common complaint and accounts for up to 10% of all emergency department (ED) visits.^1^ Acute flank pain and renal colic account for nearly 40% of all patients admitted with an acute abdomen.^2^ There are several possible causes for acute flank pain including urinary stones, pyelonephritis, muscular- or skeletal complaints and nonspecific abdominal pain.^2^

The European Association of Urology (EAU) Guidelines provide no clear recommendations regarding diagnostic imaging in the setting of uncomplicated acute flank pain.^3^ On the other hand, immediate imaging with non-contrast computed tomography (CT) is recommended in patients who present with fever, have a solitary kidney, or when the diagnosis is uncertain.^3^ The importance of immediate CT in acute management is debated.^4–6^ However, the main argument for performing an acute CT scan is to enable early diagnosis and ensure prompt intervention in patients with obstruction and infection.^7,8^ In addition, follow-up can be omitted in patients when no stones are detected on immediate CT imaging. Counterarguments state that acute CT is seldom necessary in cases with typical clinical presentation and symptoms, and that immediate imaging may lead to unnecessary early surgical interventions.

At Haukeland University Hospital (HUH), immediate diagnostic imaging in patients admitted with typical uncomplicated acute flank pain is not considered to be indicated. Instead, non-contrast CT is deferred to an outpatient follow-up at 3–4 weeks. The rationale is that deferring immediate CT in uncomplicated acute flank pain limits patient radiation exposure, reduces the number of diagnostic examinations and avoids redundant treatments. Furthermore, the literature suggests no advantages for acute ureteroscopic (URS) stone treatment compared to deferred treatment regarding stone free status, complications and the need for auxiliary procedures.^9^

To advance the debate, we reviewed records of patients admitted to the ED at HUH with acute flank pain. Our objectives were to quantify the use of immediate CT, evaluate its effect on treatment and identify predictors of intervention to determine which patients would gain the greatest benefit from acute CT.

## Materials and methods

### Study population and setting

As part of a clinical audit, a retrospective review of the medical records was performed for patients referred to the ED at HUH in Norway with a diagnosis of acute flank pain in the period June 2023–June 2024. Outcomes of interest included clinical characteristics and pain duration before referral; need for acute CT and radiographic findings; indications for acute or deferred intervention; length of stay; rates of outpatient management and hospitalisation; readmission rates; and radiographic findings at follow-up. The American Society of Anaesthesiologists (ASA) score was used to assess the patients’ overall physical status.

Patients were categorised as having uncomplicated or complicated acute flank pain based on their clinical presentation. *Uncomplicated* acute flank pain was defined as patients with typical colic flank pain, normal creatinine levels and no signs of infection, whereas *complicated* acute flank pain was characterised by *either* CRP above 50 mg/L, creatinine levels above 130 μmol/L *or* fever (defined as temperature ⩾ 38^4^ °C). Objective measures of pain (i.e. visual analogue score) were not employed; instead, pain was assessed by each doctor in consultation with the patient. A similar individual evaluation was applied when documenting pain characteristics in the medical records.

At HUH, immediate CT is routinely reserved for patients with complicated acute flank pain only. In addition, patients who do not respond adequately to standard analgesia with NSAIDs (Diclofenac 75 mg i.m. ×2) and an additional dose of morphine, are also examined with acute CT imaging. However, because clinical assessment was subjective, the decision to perform acute CT was ultimately made by the examining doctor in each case.

Indications for acute drainage of the collecting system were infection or renal impairment due to obstruction. Immediate URS was performed in the cases of ureteric stones due to intractable pain or markedly elevated creatinine levels. The decision to intervene remained consistent throughout the entire period.

A non-contrast CT was routinely scheduled 3–4 weeks later for all patients without CT at admission and for those with stones expected to pass spontaneously. Patients with persistent ureteral stones at follow-up were referred for definitive treatment.

### Statistics

The results are reported as absolute numbers and as means or medians with corresponding interquartile ranges. Independent samples *t-*tests were performed comparing continuous variables, such as duration of hospital stay and creatinine levels for patients undergoing acute CT and for those who did not. Associations between categorical variables, such as performing acute CT or the need for intervention according to the clinical categories of flank pain, were assessed using exact chi-squared tests or Fisher′s exact tests. Multivariable logistic regression was performed to identify factors predictive of intervention. Statistical analyses were conducted using IBM SPSS Statistics 29.0.2.0 (IBM, Armonk, NY). A *p*-value of <0.05 was considered statistically significant.

### Ethics and approvals

The study was registered in HUH’s database for science (eProtocol, project ID 5312). As it was planned as a clinical audit, it was exempted from further ethical approval in accordance with Norwegian regulations and patient consent was not required.

## Results

Between June 1, 2023, and June 1, 2024, a total of 445 patients were referred to the ED with a diagnosis of acute flank pain. After excluding 156 patients whose flank pain had an already documented cause at the time of referral, 289 patients with 319 referrals were included in the study. There were 142 women and 147 men with a median age of 48 years (IQR 35–60). Median ASA-score was 2. Details regarding pain characteristics and clinical assessment are provided in Table 1.

**Table 1.** Pain characteristics and clinical assessment.

Characteristic	Number of patients (%)
Pain characteristic	
Constant	58 (18%)
Intermittent/colic	150 (47%)
Not specified	111 (35%)
Pain duration	
0–12 h	97 (30%)
13–24 h	59 (19%)
1–2 days	42 (13%)
3–7 days	75 (24%)
>7 days	45 (14%)
Not specified	1 (-)
Urge to move	
Yes	118 (37%)
No	36 (11%)
Not specified	165 (52%)
Pain radiation	
Yes	164 (51%)
No	50 (16%)
Not specified	105 (33%)
Nausea and vomiting	
Yes	146 (46%)
No	58 (18%)
Not specified	115 (36%)
Fever (temp ⩾ 38^4^ °C)	
Yes	7 (2%)
No	281 (88%)
Not specified	31 (10%)
Urine dip stick	
Negative	30 (9%)
Microhaematuria	160 (50%)
Nitrite and/or leucocytes	54 (17%)
Not specified or not applicable	75 (24%)

In total, 218 patients (68%) presented with typical flank pain, normal creatinine levels and no signs of infection. These patients were categorised as having *uncomplicated* acute flank pain. In contrast, 75 patients (24%) were classified as having *complicated* acute flank pain with *either* a CRP above 50 mg/L, creatinine levels above 130 μmol/L *or* fever. The remaining 26 patients (8%) could not be categorised as uncomplicated or complicated due to insufficient registration of clinical data in their medical records.

An acute CT scan was performed in 197 cases (62%) and was more commonly performed in those with complicated flank pain (68 of 75 patients [91%]) compared to those with uncomplicated flank pain (121 of 218 patients [56%]), *p* < 0.001. The diagnostic findings of acute CT are listed in Table 2. A cause of flank pain was determined in 159 cases (81%), with ureteral stone(s) being the most common finding, accounting for 122 cases (62%).

**Table 2.** Diagnosis based on acute CT (n = 197).

Diagnosis	Number of patients (%)
Negative/no pathology	35 (18%)
Ureteral stone	122 (62%)
Renal stone	13 (6.5%)
Pyelonephritis	3 (1.5%)
Biliary stone	1 (0.5%)
Diverticulitis	3 (1.5%)
Subileus	1 (0.5%)
Ileus	1 (0.5%)
Other	18 (9%)
Hydronephrosis	8 (4%)
Bladder stone	1 (0.5%)
Compression fracture Th12	1 (0.5%)
Lumbar prolapse	1 (0.5%)
Retroperitoneal metastases	1 (0.5%)
Uterus myoma	1 (0.5%)
Torqued adnexa	1 (0.5%)
Renal cancer	1 (0.5%)
Ureteral tumour	1 (0.5%)
Urinary retention	1 (0.5%)
Inflammatory inguinal process	1 (0.5%)

Characteristics of patients with ureteral stone(s) detected at acute CT are given in Table 3. Stones located in the proximal ureter were more likely to require acute intervention than distal stones, 23 of 26 proximal stones (89%) versus 41 of 83 distal stones (49%), *p* < 0.001. In addition, stones needing intervention were larger than those managed conservatively, 6 mm (IQR 4–7) versus 4 mm (IQR 3–5), *p* < 0.001.

**Table 3.** Characteristics of patients with ureteral stones detected on acute CT (*n* = 122).

Characteristics	All	No intervention	Intervention	*p*-Value
Sex, *n* (%)				
Men	79 (65%)	33 (70%)	46 (61%)	0.3
Women	43 (35%)	14 (30%)	29 (39%)	
Fever, *n* (%)				
Yes	3 (3%)	1 (2%)	2 (3%)	0.7
No	111 (97%)	42 (98%)	69 (97%)	
CRP, mean (IQR) mg/L	37 (2–43)	9 (1–9)	54 (3–74)	<0.001
Creatinine, mean (IQR) μmol/L	105 (81–123)	97 (75–109)	111 (83–131)	0.028
eGFR, mean (IQR) mL/min/1.73m^2^	70 (52–85)	77 (63–94)	66 (48–82)	0.007
Hydronephrosis, *n* (%)				
Yes	105 (86%)	38 (81%)	67 (89%)	0.3
No	17 (14%)	9 (19%)	8 (11%)	
Stone size, mean (IQR) mm	5 (3–6)	4 (3–5)	6 (4–7)	<0.001
Stone location, *n* (%)				
Upper ureter	26 (21%)	3 (6%)	23 (31%)	<0.001
Mid ureter	13 (11%)	3 (6%)	10 (13%)	
Lower ureter	83 (68%)	41 (88%)	42 (56%)	

Acute URS was performed in 48 (39%) of the ureteral stone patients, while 27 (22%) were drained with a JJ-stent or nephrostomy tube only. The remaining 47 patients (39%) with ureteral stone(s) were managed conservatively.

Considering all patients, regardless of the cause of flank pain or diagnostic imaging, 234 (73%) received conservative treatment only. Furthermore, patients who required intervention had higher CRP levels (52 vs 15 mg/L, *p* < 0.001) and creatinine levels (108 vs 87 μmol/L, *p* < 0.001) compared to those managed conservatively. Conversely, consistent with the higher creatinine, the intervention group had a lower estimated glomerular filtration rate (eGFR) than the patients managed conservatively (67 vs 86 mL/min/1.73m^2^, *p* < 0.001). In addition, complicated acute flank pain (*n* = 40 [53%]) was associated with a higher incidence of intervention than uncomplicated flank pain (*n* = 43 [20%]), *p* < 0.001.

Multivariable logistic regression was performed to identify factors predictive of intervention prior to diagnostic imaging (Table 4). In this analysis, a CRP ⩾ 60 mg/L and a creatinine level ⩾ 110 µmol/L were the only significant predictors of intervention.

**Table 4.** Multivariable logistic regression analysis predicting intervention prior to diagnostic imaging.

Risk factors	OR	95% CI for OR	*p*-Value
Patient related factors			
Sex (male vs female)	0.8	04–1.6	0.6
Age (⩾50 years vs <50 years)	1.2	0.6–2.3	0.6
Factors related to clinical findings			
Pain duration (⩽24 h vs >24 h)	0.6	0.3– 1.1	0.1
Fever (*t* ⩾ 38^4^ °C vs *t* < 38^4^ °C)	1	0.9–1	0.6
CRP (⩾50 vs <50 mg/L)	2.4	1.1–5.0	0.024
Creatinine (⩾110 vs<110 μmol/L)	3.6	1.4–9.2	0.009
eGFR (⩽60 vs>60 mL/min/1.73m^2^)	1.4	0.6–3.4	0.5

The median hospital stay was longer in patients who underwent an acute CT scan (2 days, IQR 1–2) than in those in whom it was omitted (0 days, IQR 0−0), *p* < 0.001. In total, 112 (35%) of the 319 admissions were discharged from the emergency department after a median of 3 h (IQR 2–4) and were managed as outpatients. Uncomplicated acute flank pain was more common among those treated as outpatients than among patients overall. Among the cases managed as outpatients, 103 (92%) presented with uncomplicated acute flank pain, while 9 (8%) had complicated acute flank pain. In addition, only 10 outpatients (9%) underwent acute CT scan, and acute intervention was not needed at all in the outpatient group. Sixteen outpatients (14%) needed readmission, four of these due to infection and the remaining because of pain.

In total, 219 patients (69%) underwent CT at follow-up after 3–4 weeks, and a persistent ureteral stone requiring *elective* ureteroscopy was registered in 40 (19%). Twenty-six patients (8%) had no CT scan at emergency admission or at follow-up.

## Discussion

A non-contrast CT is considered the first-choice diagnostic imaging method in patients with acute flank pain.^3,10^ However, the diagnostic value and therapeutic impact of immediate CT in acute flank pain remain uncertain. In this study, we have examined clinical characteristics and outcomes for patients with acute flank pain admitted to our institution over a 12-month period. In addition, we have investigated the impact of immediate CT on the need for acute intervention.

Categorising acute flank pain patients as uncomplicated or complicated based on clinical assessment helps identify those patients who need immediate diagnostic imaging and possibly acute intervention. In the present study, 68% of the patients could be categorised as having uncomplicated acute flank pain, defined by colic pain, normal CRP and creatinine levels, and no signs of infection. By comparison, 24% of the patients were categorised as having complicated acute flank pain with CRP > 50 mg/L, creatinine levels > 110 µmol/L or fever. The remaining 8% could not be categorised due to insufficient registration of clinical data in their medical records.

At HUH, immediate diagnostic imaging in patients with uncomplicated acute flank pain has been considered unnecessary, and CT is postponed to follow-up 3–4 weeks later. This strategy is intended to reduce patients’ radiation exposure, decrease the number of diagnostic examinations and potentially avoid unnecessary treatments. Conversely, an immediate CT scan should be performed in cases of complicated flank pain, as recommended by the EAU Guidelines.^3^ Despite the current protocol at our institution, a total of 197 patients (62%) had an immediate CT scan, 121 of 218 (56%) with uncomplicated flank pain and 68 of 75 (91%) with complicated flank pain. Although a CT was performed at a significantly higher rate among the complicated patients, the high number of CTs in the uncomplicated patients may reflect physicians’ reluctance to rely solely on their clinical acumen. This is supported by the study of Meyer et al., in which low physician confidence was associated with an increased number of diagnostic tests.^11^

A cause of flank pain was determined in 159 cases (81%), and a ureteral stone was the most common finding, accounting for 122 cases (62%). These numbers are higher than those reported in other studies reporting findings of immediate CT in acute flank pain, where a detected cause ranges from 50% to 64% and ureteral stones are registered in 41%–50%.^7,12,13^ The differences may, at least partly, be explained by the organisation of the healthcare systems in different countries. Patients with acute flank pain admitted to EDs in Norway, have already been assessed in the primary healthcare service and/or a minor injuries unit. In other countries, the healthcare systems may be organised differently, and the patients may present directly to EDs without prior referral from a general practitioner, which may result in a less selected patient population.^14^ Sweden is among the countries with the latter type of healthcare organisation. Despite a less selected population assessed in the ED, 87% of the patients who underwent immediate CT were diagnosed with a ureteral stone in the study by Utter et al.^8^ In the same study, 57% of patients were discharged from the ED after clinical assessment without acute CT, illustrating an approach to acute flank pain similar to that in our hospital.^8^

The main purpose of performing immediate CT in patients with acute flank pain is to identify those who need acute intervention. In the present study, a total of 85 patients (27%) underwent acute intervention, regardless of the cause of flank pain. On further investigation, these patients had significant higher levels of CRP, creatinine and correspondingly lower eGFR compared with those managed conservatively. In addition, being categorised with complicated acute flank pain was associated with a significantly higher incidence of intervention than inpatients with uncomplicated flank pain. The results illustrate that patients with acute flank pain who require further diagnostic imaging can be identified based on clinical assessment and simple blood tests, rather than by performing immediate CT in all cases. Furthermore, looking at the patients diagnosed with ureteral stones (*n* = 122), acute URS was performed in 48 patients (39%), 27 (22%) were drained with a JJ-stent or nephrostomy tube only, and the remaining 47 patients (39%) were treated conservatively. Similarly, ureteral stone patients who required acute intervention had significantly higher levels of CRP and creatinine as well as significantly lower eGFR than those treated conservatively.

Clinical nomograms have been developed to help predict the diagnosis of ureteral stones without the use of CT.^15,16^ The STONE score consists of five clinical factors that can yield a maximum of 13 points: sex, duration of pain, ethnicity, nausea or vomiting and presence of microhaematuria. In Moore et al.’s study, the presence of a ureteral stone on non-contrast CT was 9.2% in the low probability group (score 0–5), 51.3% in the moderate probability group (score 6–9) and 88.6% in the high probability group (score 10–13).^15^ Fukuhara et al. improved the sensitivity of clinical assessment by adding the presence of hydronephrosis on ultrasound to their CHOKAI score. In total, up to 13 points can be achieved in the CHOKAI score based on sex, age, duration of pain, nausea or vomiting, presence of microhaematuria, previous history of urolithiasis and the presence of hydronephrosis. Using a cut-off score of 6, the CHOKAI score achieved a sensitivity for ureteral stone of 0.93.^16^ Furthermore, in the study by Fukuhara et al., CHOKAI scores of 10–13 were only achieved for patients with a ureteral stone.

To note, none of the scoring systems include pain characteristics such as colic pain, urge to move and pain radiation. These are all symptoms described typical for acute ureteral stone attack.^17^ The presence of such symptoms could therefore aid in the clinical decision-making in patients with acute flank pain. In addition, including CRP, creatinine and eGFR could improve the accuracy of the clinical assessment when determining which patients would benefit most from an immediate CT.

In total, 26 patients (8%) had neither a CT scan at emergency admission nor at follow-up. The reason for this is not known and these patients are at risk for being lost to further treatment of follow-up.

The present study has several limitations. The retrospective design may have impeded complete data collection for all patients. Insufficient registration of clinical data in the medical records prevented 26 patients (8%) from being categorised as uncomplicated or complicated. Furthermore, no validated tool for pain assessment was employed in this study. The assessment of pain and symptoms was therefore based solely on patients′ accounts to the physician and the corresponding documentation. This lack of standardisation is a clear limitation of the study but does reflect real world practice. For the same reason, pain was not included as a factor to predict intervention. In addition, we did not assess patients’ use of complementary or alternative therapies, which may influence symptom perception and healthcare-seeking behaviour.^18^

## Conclusion

In the present study, immediate CT was performed in 62% of patients with acute flank pain admitted to the ED. Acute intervention was required in only 27% of all patients. Focusing on complete clinical investigation and categorising patients as having uncomplicated or complicated acute flank pain may help identify who would benefit from acute CT imaging and predict the need for intervention. Immediate CT in patients with acute flank pain may be reserved for complicated cases with signs of infection, elevated CRP and creatinine levels. In uncomplicated cases CT can safely be deferred to follow-up after 3–4 weeks, which may lead to a shorter treatment course for patients in the ED.
